# Saroglitazar Versus Simvastatin for Metabolic and Alcohol-Associated Liver Disease (MetALD)

**DOI:** 10.7759/cureus.85652

**Published:** 2025-06-09

**Authors:** Anand Kulkarni, Zaid M Gangdany, Ramyashree Ramagundam, Mithun Sharma, Nagaraja R Padaki

**Affiliations:** 1 Hepatology, Asian Institute of Gastroenterology, Hyderabad, IND; 2 Hepatology, Asian Institute of Gastroenterology (AIG) Hospitals, Hyderabad, IND

**Keywords:** alcohol-related liver disease, dyslipidemia, masld, metald, transient elastography

## Abstract

Individuals with steatotic liver disease who consume significant amounts of alcohol and meet at least one cardiometabolic criterion are classified as having metabolic and alcohol-associated liver disease (MetALD). The efficacy of saroglitazar and simvastatin in this population remains unclear. In this single-center retrospective cohort study, 102 patients with MetALD were included. The reduction in CAP score was greater in the saroglitazar group (-40 (-109 to 3) dB/m) compared to the simvastatin group (-33 (-100 to 36) dB/m), although this difference did not reach statistical significance (P = 0.08). However, a significant difference was observed in the change in liver stiffness measurement (LSM) scores, with the saroglitazar group showing a greater reduction (-1.9 (-19.5 to 26.3) kPa) than the simvastatin group (-0.8 (-12.2 to 9) kPa) (P = 0.01). Saroglitazar also demonstrated a more pronounced effect on glycosylated hemoglobin (HbA1c), with a median decrease of -0.61 ± 0.96 compared to -0.1 ± 0.4 in the simvastatin group (P = 0.02). Saroglitazar is more effective than simvastatin in reducing CAP, LSM, and HbA1c over six months. Further prospective, well-controlled randomized studies are warranted to validate these findings.

## Introduction

Steatosis is a common feature of both alcohol-related liver disease and metabolic dysfunction-associated steatotic liver disease (MASLD) [1,2]. Individuals with steatotic liver disease who meet at least one cardiometabolic criterion and consume alcohol in the range of 140-350 g/week for women or 210-420 g/week for men are classified as having metabolic and alcohol-associated liver disease (MetALD) [1]. Treatment for these patients typically involves managing cardiometabolic risk factors and promoting strict alcohol abstinence. However, no clinical trials to date have evaluated pharmacological treatments specifically for this condition [3]. A large UK Biobank study recently reported that the lipid profiles of patients with MetALD and MASLD are largely comparable [4]. As a result, lipid-lowering agents, particularly HMG-CoA reductase inhibitors, are commonly prescribed for these populations. Simvastatin, in particular, has shown benefits in patients with MASLD in a few small cohort studies [5].

Saroglitazar is a peroxisome proliferator-activated receptor (PPAR)-α/γ agonist that modulates lipid and glucose metabolism pathways. It has demonstrated efficacy in treating diabetic dyslipidemia and metabolic dysfunction-associated steatohepatitis (MASH) [6,7]. Moreover, PPAR-α/γ agonists have been shown to reduce alcohol intake in preclinical studies, although this effect has not been evaluated in clinical settings [8]. Saroglitazar is approved by the Drug Controller General of India for both diabetic dyslipidemia and MASH, and it has been granted fast-track designation by the US FDA for primary biliary cholangitis [9,10]. However, to our knowledge, no studies have evaluated the efficacy of saroglitazar or statins in patients with MetALD - an evidence gap this study aims to address.

## Materials and methods

In this single-center retrospective study, all consecutive patients diagnosed with MetALD who received either saroglitazar or simvastatin were included. The primary objective was to evaluate the therapeutic effects of both drugs on the controlled attenuation parameter (CAP) score and liver stiffness measurement (LSM) using transient elastography (TE) via FibroScan^®^ (Echosens, Paris, France). Secondary objectives included comparing changes in fibrosis 4 (Fib-4) scores, lipid profiles, and glycosylated hemoglobin (HbA1c) levels after six months of treatment. The study also assessed and compared weight loss outcomes and alcohol relapse rates between the two treatment groups.

Patients were selected through a comprehensive review of medical records in the hospital information system. Inclusion criteria were a diagnosis of MetALD and treatment with either simvastatin or saroglitazar. Patients with a prior history of hepatic decompensation were excluded to ensure only those with stable liver function were analyzed.

Baseline data, including demographic information, clinical characteristics, and initial laboratory values, were collected. Demographic variables included age, sex, BMI, and relevant medical history. Biochemical variables included alanine transaminase (ALT), aspartate transaminase (AST), total bilirubin, and albumin. All patients underwent TE for liver stiffness and steatosis assessment using the FibroScan^®^ device, performed according to manufacturer guidelines. Valid results required at least 10 measurements with an interquartile range of less than 20%. CAP scores were used to quantify hepatic steatosis, while TE scores represented liver fibrosis.

Patients with missing or incomplete data on liver function, alcohol use, or clinical outcomes were excluded. Lipid profile parameters - total cholesterol, triglycerides, low-density lipoprotein (LDL), and high-density lipoprotein (HDL) - were recorded. HbA1c levels were also monitored as an indicator of glycemic control. Changes in these metabolic parameters were analyzed to evaluate drug efficacy in the context of MetALD.

Alcohol consumption history was assessed and quantified in grams per week based on the type and volume of alcohol consumed [11]. Alcohol use was also tracked during follow-up visits to identify relapse, and relapse rates were compared between treatment groups.

Statistical analysis

All analyses were conducted using IBM SPSS Statistics for Windows, Version 29.0 (Released 2022; IBM Corp., Armonk, NY, USA). Continuous variables were expressed as mean ± SD or median (range), depending on data distribution. Student’s t-test was used to compare normally distributed variables, while the Mann-Whitney U test was used to compare nonparametric data. Categorical variables, including sex and alcohol relapse, were compared using either Pearson’s chi-square or Fisher’s exact test, depending on the distribution and expected frequencies. A p-value <0.05 was considered statistically significant.

## Results

A total of 102 patients were included, all male, with a mean age of 42.1 ± 9.4 years and a mean BMI of 25.8 ± 3.9 kg/m². The mean duration of alcohol use was 16.5 ± 7.3 years, with a mean intake of 291.2 ± 71 g/week. The cohort had a median CAP score of 287.5 dB/m (range: 170-400) and a median LSM of 9.1 kPa (range: 4-30); approximately 45% (46/102) had LSM >10 kPa, indicative of significant fibrosis.

Fifty-seven patients were treated with saroglitazar, and 45 received simvastatin. All saroglitazar-treated patients received a 4 mg dose. Among simvastatin users, four received 20 mg/day and the remainder 10 mg/day, with a treatment duration of six months in both groups.

Baseline characteristics - including CAP and LSM scores, as well as lipid profile parameters - were comparable between the two groups. A similar proportion of patients in both groups met the cardiometabolic criteria (Table 1).

**Table 1:** Baseline variables among the included patients ALP, alkaline phosphatase; ALT, alanine transaminase; AST, aspartate transaminase; CAP, controlled attenuation parameter; DM, diabetes mellitus; FBS, fasting blood sugar; Fib-4, fibrosis 4; HDL, high-density lipoprotein; INR, international normalized ratio; LDL, low-density lipoprotein; LSM, liver stiffness measurement; MetALD, metabolic and alcohol-associated liver disease; VLDL, very-low-density lipoprotein

Variable	Saroglitazar (n = 57)	Simvastatin (n = 45)	Test statistic	P-value
Age (years)	41 (26-64)	42 (22-66)	U = 1213.9	0.63
Men (%)	100%	100%	-	-
BMI (kg/m²)	25.15 (16.61-38.51)	25.4 (17.2-34.1)	U = 1152.5	0.38
Dose of drug (mg/day)	4	10 (10-20)	-	-
DM, n (%)	21 (36.8%)	19 (42.2%)	χ² = 0.3	0.58
Hypertension, n (%)	9 (15.8%)	12 (26.7%)	χ² = 1.8	0.17
Hypothyroidism, n (%)	4 (7.0%)	1 (2.2%)	χ² = 1.2	0.38
MetALD criteria, n (%)			χ² = 3.5	0.47
1	3 (5.3%)	3 (6.7%)		
2	9 (15.8%)	9 (20.0%)		
3	13 (22.8%)	4 (8.9%)		
4	23 (40.4%)	21 (46.7%)		
5	9 (15.8%)	8 (17.8%)		
Alcohol intake (g/week)	282.9 ± 66.3	301.6 ± 76.0	U = 1111.5	0.24
Total bilirubin (mg/dL)	1.2 (0.5-23.4)	1.0 (0.5-34.5)	U = 1005.0	0.1
Direct bilirubin (mg/dL)	0.3 (0.1-14.5)	0.2 (0.1-17.0)	U = 1054.5	0.16
AST (U/L)	65 (23-600)	58.5 (16-308)	U = 1093.5	0.27
ALT (U/L)	60 (18-272)	53.5 (16-380)	U = 1147.5	0.46
ALP (U/L)	103 (54-292)	105 (60-190)	U = 1233.0	0.88
Albumin (g/dL)	4.3 (2.5-5.2)	4.35 (2.6-5.2)	U = 1105.5	0.68
INR	0.98 (0.82-2.33)	0.98 (0.86-2.41)	U = 1059.5	0.92
FBS (mg/dL)	97.5 (74-274)	101 (80-305)	U = 966.0	0.11
HbA1c (%)	5.8 (4.3-12.3)	5.9 (5.0-10.8)	U = 1088.0	0.49
Hemoglobin (g/dL)	14.1 (6.9-17.6)	14.2 (8.5-17.9)	U = 1171.0	0.57
Creatinine (mg/dL)	0.85 (0.67-1.93)	0.84 (0.6-1.19)	U = 1193.5	0.78
Total cholesterol (mg/dL)	213 (73-342)	215 (123-316)	U = 1254.0	0.84
HDL (mg/dL)	45 (10-148)	48 (18-103)	U = 1152.5	0.38
VLDL (mg/dL)	41 (11-116)	41 (15-149)	U = 1260.5	0.88
LDL (mg/dL)	130 (36-219)	124 (70-229)	U = 1221.5	0.68
Triglycerides (mg/dL)	207 (57-871)	207 (77-743)	U = 1239.5	0.77
CAP (dB/m)	288 (195-400)	287 (170-381)	U = 1196.0	0.56
LSM (kPa)	8.9 (4.5-30.0)	9.8 (4.0-25.0)	U = 1214.0	0.64
LSM >10 kPa, n (%)	24 (42.1%)	22 (49.0%)	χ² = 0.46	0.49
Fib-4 score	1.9 (0.56-16.4)	1.6 (0.5-11.6)	U = 1171.0	0.57
Duration of therapy (months)	6 (2-6)	6 (1-6)	U = 1133.0	0.16

The reduction in CAP score was greater in the saroglitazar group (-40 (-109 to 3) dB/m) compared to the simvastatin group (-33 (-100 to 36) dB/m), although the difference was not statistically significant (P = 0.08). However, a significant difference was observed in the change in LSM scores between the saroglitazar group (-1.9 (-19.5 to 26.3) kPa) and the simvastatin group (-0.8 (-12.2 to 9) kPa) (P = 0.01). By the end of therapy, there was no significant difference in Fib-4 scores between the two groups (saroglitazar: -0.54 ± 2.28 vs. simvastatin: -0.15 ± 1.48; P = 0.46). Changes in lipid profile parameters and liver enzymes (AST and ALT) were also comparable between the groups (Table 2). Saroglitazar demonstrated a more pronounced effect on glycemic control, with a median reduction in HbA1c of -0.1 (-3.6 to 0), compared to simvastatin, which showed a minimal change of 0 (-1.2 to 0.8) (P = 0.02).

**Table 2:** Comparison of the delta change in each variable between the two groups Follow-up lipid profile assessments were available for 34 patients in the saroglitazar group and 22 patients in the simvastatin group. Follow-up HbA1c measurements were available for 33 patients in the saroglitazar group and 21 in the simvastatin group. Changes in Fib-4 scores (dFib-4) were calculated for 41 patients in the saroglitazar group and 23 patients in the simvastatin group. ALT, alanine transferase; AST, aspartate transferase; CAP, controlled attenuation parameter; d, delta; Fib-4, fibrosis 4; HDL, high-density lipoprotein; LDL, low-density lipoprotein; LSM, liver stiffness measurement; TC, total cholesterol; TG, triglycerides; VLDL, very-low-density lipoprotein

Variable	Saroglitazar	Simvastatin	Test statistic	P-value
dCAP (dB/m)	-40 (-109 to 3)	-33 (-100 to 36)	U = 1027.5	0.08
dLSM (kPa)	-1.9 (-19.5 to 26.3)	-0.8 (-12.2 to 9)	U = 918.0	0.01
dFib-4 score	-0.54 (-9.04 to 2.95)	0.5 (-4.07 to 1.36)	U = 406.0	0.35
dAST (U/L)	-4 (-177 to 120)	6 (-287 to 44)	U = 522.5	0.76
dALT (U/L)	-8.5 (-149 to 62)	-15 (-350 to 59.7)	U = 507.5	0.63
dTotal cholesterol (mg/dL)	-24 (-197 to 64)	-25.5 (-144 to 140)	U = 338.5	0.54
dHDL (mg/dL)	0 (-50 to 20)	0 (-24 to 30)	U = 373.0	0.98
dVLDL (mg/dL)	-3 (-59 to 12)	0 (-60 to 10)	U = 337.5	0.52
dLDL (mg/dL)	-15.5 (-133 to 88)	-10 (-300 to 49)	U = 361.5	0.83
dTg (mg/dL)	-44 (-373 to 59)	-7 (-300 to 49)	U = 297.5	0.19
dHbA1c (%)	-0.1 (-3.6 to 0)	0 (-1.2 to 0.8)	U = 223.0	0.02

Two patients in the saroglitazar group reported mild adverse effects, including fatigue, itching, and abdominal discomfort, whereas no adverse events were reported in the simvastatin group. Within six months, 15 patients (26%) in the saroglitazar group and 14 patients (31.1%) in the simvastatin group experienced at least 5% weight loss (P = 0.59). Alcohol relapse occurred in 28% of patients in the saroglitazar group and 31.1% in the simvastatin group (P = 0.73).

## Discussion

The key findings from this study suggest that saroglitazar can reduce hepatic steatosis and may also reduce fibrosis in patients with MetALD. The median CAP and LSM values indicated that the cohort had moderate hepatic steatosis, with a substantial proportion (45%) showing liver stiffness values greater than 10 kPa, consistent with advanced fibrosis.

Saroglitazar also led to a greater reduction in HbA1c levels compared to simvastatin, consistent with its known effects on glucose metabolism. This may be attributed to its dual PPAR agonist mechanism, which directly targets both lipid and glucose metabolic pathways, potentially making it a more suitable therapeutic option for MetALD patients with metabolic dysregulation [6]. Simvastatin, by contrast, has been shown to improve microcirculatory function in MASLD by downregulating oxidative and advanced lipoxidation end product-receptor for advanced glycation end products stress, thereby improving steatosis, fibrosis, and inflammatory markers [12]. However, in this study, its effects on CAP and HbA1c were less pronounced than those of saroglitazar, consistent with previous findings [6,7]. The limited impact of simvastatin on HbA1c is expected, given that statins primarily modulate lipid metabolism and exert minimal influence on glucose regulation.

Both treatment groups exhibited only modest reductions in liver stiffness, suggesting that while saroglitazar and simvastatin may help mitigate steatosis, their capacity to reverse fibrosis over a six-month period is limited, although saroglitazar did achieve a statistically significant reduction in LSM [7,13]. These findings underscore the need for longer-term studies incorporating histological assessment to determine the potential of these agents to halt or reverse fibrosis progression [14]. In clinical practice, saroglitazar is typically prescribed for patients with diabetes and dyslipidemia, whereas simvastatin is favored in those with evidence of portal hypertension, although no prespecified selection criteria were applied in this study.

Both groups showed similar improvements in lipid parameters, including total cholesterol, LDL, HDL, and triglycerides. Despite saroglitazar’s known lipid-lowering effects, the comparable changes observed in both groups suggest that its principal benefit in MetALD may lie in glycemic control rather than lipid regulation.

Relapse rates were similar between the groups, indicating that neither drug had a significant impact on modifying alcohol consumption behavior. This finding highlights the need to complement pharmacologic therapy with behavioral interventions to support sustained abstinence in MetALD. Moreover, the lack of impact of PPAR-µ/g agonists on alcohol relapse reinforces findings from earlier preclinical studies [8,15]. Pharmacological treatments for alcohol use disorder, which were not assessed in this study, may be necessary for addressing this component of the disease.

The coexistence of metabolic dysfunction in patients with alcohol use disorder is associated with an elevated risk of fibrosis, and ongoing alcohol intake further accelerates fibrosis progression [16]. Consequently, neither saroglitazar nor simvastatin alone proved effective in reversing fibrosis in this setting.

Both treatments were generally well tolerated. Mild adverse effects - fatigue, itching, and abdominal discomfort - were reported by two patients in the saroglitazar group, while no adverse events were reported among those receiving simvastatin. Importantly, these side effects were mild and did not lead to treatment discontinuation. Saroglitazar has an established safety profile and has not been associated with hepatotoxicity, a finding consistent with this study [17].

Limitations

Several limitations should be acknowledged. This was a retrospective study with a relatively small sample size and a follow-up period of only six months, which may not have been sufficient to detect meaningful changes in fibrosis or long-term clinical outcomes. Additionally, lifestyle factors such as diet and physical activity, which could influence metabolic outcomes and weight loss, were not accounted for.

## Conclusions

This study suggests that saroglitazar is more effective than simvastatin in reducing hepatic steatosis, liver stiffness, and HbA1c levels in patients with MetALD. While both treatments had similar effects on lipid profiles, liver stiffness, and weight loss, the added glycemic benefit of saroglitazar supports its potential use in MetALD patients with metabolic dysregulation and dyslipidemia.

Prospective, randomized studies with larger cohorts and extended follow-up are needed to validate these findings. Future research should also explore optimal dosing strategies and evaluate the combined effects of pharmacotherapy and lifestyle modification on alcohol use, liver health, and cardiometabolic outcomes in MetALD.
